# Undifferentiated Thyroid Carcinoma Caused Sudden Airway Obstruction

**DOI:** 10.5811/westjem.2015.10.28836

**Published:** 2015-12-01

**Authors:** Yudai Iwasaki

**Affiliations:** Ohta Nishinouchi Hospital, Department of Critical Care and Emergency Medicine, Fukushima, Japan

## Case report

**Figure 1:**
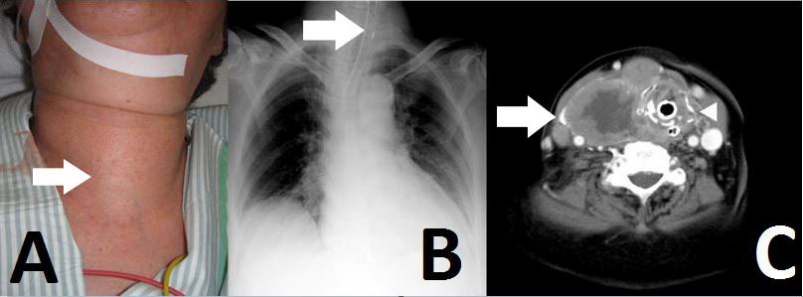
(A) Image showing swelling of the neck in our patient (arrow). (B) Chest radiograph after intubation showing a shift of the trachea to the left (arrow). (C) Contrast computed tomography image showing the thyroid tumor superior to the right lobe (arrow) and tumor invasion into the trachea at the height of the cricoid cartilage (arrow head).

An 81-year-old woman was admitted to our emergency department (ED) with neck swelling (Figure 1A) and advancing dyspnea. Stridor was noted on auscultation of her neck, and her breathing was labored. We immediately diagnosed airway obstruction, and emergency intubation was performed using a video laryngoscope (AWS-S100L®, Pentax Corporation, Tokyo, Japan). The epiglottis was found to have shifted to the left on chest video images and chest radiograph (Figure 1B). After intubation, computed tomography and cervical ultrasonography were performed, and we noted swelling of the thyroid, which was superior to the right lobe, and tumor invasion into the trachea without lung metastases (Figure 1C). After admission, fine needle aspiration was performed, and she was diagnosed with undifferentiated carcinoma. We could not perform tracheostomy or place an intratracheal stent because of continuous intratracheal bleeding and disseminated intravascular coagulation. The patient died 28 days after admission.

## Discussion

Airway obstruction caused by thyroid carcinoma is rare.^1^,^2^ Lung metastases often are associated with respiratory symptoms and are the most fatal complication.^3^

When patients present to the ED with airway obstruction, physicians should consider acute epiglottitis, peritonsillar or retropharyngeal abscess, and foreign body aspiration as possible causes. Although invasion of thyroid carcinoma resulting in airway obstruction is rare, physicians should consider it an oncologic emergency. Visual examination, palpation, and auscultation of the neck can help in the differential diagnosis of airway obstruction.
